# Impact of Isolated High Home Systolic Blood Pressure and Diabetic Nephropathy in Patients with Type 2 Diabetes Mellitus: A 5-Year Prospective Cohort Study

**DOI:** 10.3390/jcm10091929

**Published:** 2021-04-29

**Authors:** Nobuko Kitagawa, Noriyuki Kitagawa, Emi Ushigome, Hidetaka Ushigome, Isao Yokota, Naoko Nakanishi, Masahide Hamaguchi, Mai Asano, Masahiro Yamazaki, Michiaki Fukui

**Affiliations:** 1 Department of Endocrinology and Metabolism, Graduate School of Medical Science, Kyoto Prefectural University of Medicine, Kyoto 602-8566, Japan; nobuko-s@koto.kpu-m.ac.jp (N.K.); nori-kgw@koto.kpu-m.ac.jp (N.K.); naoko-n@koto.kpu-m.ac.jp (N.N.); mhama@koto.kpu-m.ac.jp (M.H.); maias@koto.kpu-m.ac.jp (M.A.); masahiro@koto.kpu-m.ac.jp (M.Y.); michiaki@koto.kpu-m.ac.jp (M.F.); 2 Department of Endocrinology and Metabolism, Kameoka Municipal Hospital, Kyoto 621-8585, Japan; 3 Department of Organ Transplantation and General Surgery, Graduate School of Medical Science, Kyoto Prefectural University of Medicine, Kyoto 602-8566, Japan; ushi@koto.kpu-m.ac.jp; 4 Department of Biostatistics, Graduate School of Medicine, Hokkaido University, Hokkaido 060-8638, Japan; yokotai@pop.med.hokudai.ac.jp

**Keywords:** albuminuria, diabetes mellitus, isolated high home systolic blood pressure, diabetic nephropathy

## Abstract

Background: A previous 2-year cohort study has shown that isolated high home systolic blood pressure (IH-HSBP) may increase the risk of diabetic nephropathy, using normal HBP as a reference. However, this association has not been previously assessed in the medium to long term. Methods: This prospective 5-year cohort study of 424 patients, with normal or mildly increased albuminuria, investigated the effect of IH-HSBP on the risk of diabetic nephropathy in patients with type 2 diabetes mellitus. Diabetic nephropathy was defined as an advancement from normal or mildly increased albuminuira to moderate or severely increased albuminuria. Results: Among 424 patients, 75 developed diabetic nephropathy during the study period. The adjusted odds ratio for developing diabetic nephropathy given IH-HSBP was 2.39 (95% confidence interval, 1.15–4.96, *p* = 0.02). The odds ratio for developing nephropathy in patients with IH-HSBP younger than 65 years was higher than that in patients with IH-HSBP older than 65 years. Conclusion: IH-HSBP was associated with an increased risk of diabetic nephropathy among type 2 diabetes mellitus patients with normal or mildly increased albuminuria in the medium to long term. The results support and strengthen previous reports. These findings suggest that IH-HSBP might be a useful marker in disease prognostication.

## 1. Introduction

Home blood pressure (HBP) control is paramount to diabetic nephropathy prevention [1]. Several important factors of HBP, including day-to-day variability [2] or pulse pressure [3], have been reported as relevant to the risk of diabetic nephropathy.

Isolated systolic hypertension (ISH) is diagnosed when systolic blood pressure (SBP) is hypertensive, while diastolic blood pressure (DBP) is normotensive [4]. ISH has been shown to increase the risk of premature mortality in patients with cardiovascular disease; it is a common form of hypertension [5,6,7].

ISH expressed as HBP (home ISH) has also been shown to affect the risk of diabetic nephropathy. In fact, our group has previously shown that isolated high home systolic blood pressure (IH-HSBP) might be a useful marker in the prognostication of diabetic nephropathy, based on data from a 2-year cohort study [8]. Nevertheless, the follow-up period in that study was relatively short, likely limiting its statistical power. To address this limitation, we performed a follow-up study with patients diagnosed with type 2 diabetes mellitus (DM), aiming to provide a valid assessment of the impact of ISH on the risk of diabetic nephropathy in this patient group over the medium to long term.

## 2. Design and Methods

We used the same resources in our previous study, which is based on data from the HBP cohort of patients with type 2 diabetes mellitus who had regularly attended the diabetes outpatient clinic at the Kyoto Prefectural University of Medicine Hospital or other general hospitals located in Japan (KAMOGAWA-HBP study) [1].

The present study included patients with type 2 DM; the impact of HBP on the risk of diabetic nephropathy was evaluated. Nephropathy was graded as follows: normal or mild albuminuria, defined as urinary albumin/creatinine ratio (UACR) < 30 mg per gram of creatinine (mg/g Cr); moderately increased albuminuria (microalbuminuria), defined as UACR 30–300 mg/g Cr; or severely increased albuminuria (macroalbuminuria), defined as UACR >300 mg/g Cr [9,10,11,12,13,14]. The development of diabetic nephropathy was defined as an advancement from normal or mild albuminuira to moderately or severely increased albuminuria within 5 years. The study protocol was approved by the local Research Ethics Committee, RBMR-E-349; the study adhered to the principles of the Declaration of Helsinki, and informed consent was obtained from all patients prior to enrollment.

### 2.1. Data Collection

Blood samples for biochemical measurements were taken in the morning. Serum lipid profile (including levels of triglycerides, low-density lipoprotein cholesterol, and high-density lipoprotein cholesterol) and levels of creatinine and hemoglobin A1C (HbA1c), and of other biochemical markers, were assessed by standard laboratory methods. The data collection of urinary samples was performed simultaneously with the beginning of HBP measurements. An immunoturbidimetric assay was used to measure UACR; the mean value of three consecutive urinary measurements was equivalent to UACR. Levels of HbA1c were classified and reported according to the National Glycohemoglobin Standardization Program guidelines, as recommended by the Japan Diabetes Society [15]. Data on patient demographic and clinical characteristics, including sex, age, duration of DM, smoking status, and those who consumed alcohol or antihypertensive medication were collected at the same time as HBP measurements began. To measure brachial–ankle pulse wave velocity (baPWV), the volume plethysmographic method was used, which was also the method utilized in our previous cohort study [16]. Diagnosis of diabetic nephropathy was based on the Diagnostic Nephropathy Study Group criteria [17]. Alcohol drinking status (never, social, or everyday) and smoking status (never, past, or current) were checked by interview. Type 2 DM was diagnosed when a fasting plasma glucose level was more than 126 mg/dl (7.0 mmol/L), or a random plasma glucose was more than 200 mg/dl (11.1 mmol/L), based on the American Diabetes Association criteria [18].

### 2.2. HBP Measurements

Patients were instructed to measure their BP 3 times each morning and evening for 14 consecutive days, and the 14-day average of the 3 morning and 3 evening mean values were calculated for each. Patients were instructed to measure their morning BP within 1 h of waking up, before breakfast, before taking medication, having sat, and having rested for at least 5 min [19]. Similar instructions applied to evening BP measurements, which were obtained before bedtime. Eating was prohibited for over one hour before measurement before going to bed. Moreover, patients were instructed that the cuff of the measuring device should be placed around the contralateral side of the dominant arm, with its position maintained at the level of the heart. HBP measurements were performed with an automated device—HEM-70801C (Omron Healthcare Co. Ltd., Kyoto, Japan)—which used a digital display to present values of SBP/DBP and heart rate, measured using the cuff-oscillometric method. HEM-70801C uses the same components and BP-determining algorithm as those of another device, HEM-705IT, which was previously validated and satisfied the criteria of the British Hypertension Society protocol [20].

In the Japanese Society of Hypertension Guidelines for the Management of Hypertension (JSH 2019) [21], the target level of HBP control is under 125/75 mmHg in hypertensive patients with DM. Patients were classified into 4 groups based on HBP levels: normal HBP (morning SBP < 125 mmHg and morning DBP < 75 mmHg), isolated high IH-HSBP (morning SBP > 125 mmHg and morning DBP < 75 mmHg), isolated high home DBP (IH-HDBP) (morning SBP < 125 mmHg and morning DBP > 75 mmHg), and high HBP (morning SBP > 125 mmHg and morning DBP > 75 mmHg) [21,22].

### 2.3. Statistical Analysis

Participant baseline characteristics were reported as median, with interquartile range or count, as suitable. Logistic regression analysis was used to assess the relationship between IH-HSBP, IH-HDBP, and high HBP, and the risk of diabetic nephropathy, with “normal HBP” set as a reference. The following factors were included as covariates in the adjusted models: sex, body mass index (BMI), duration of diabetes, levels of HbA1c, of total cholesterol, of creatinine, and use of antihypertensive medication (Model 2). Separate adjustments were made for the use of renin–angiotensin–aldosterone system inhibitors instead of other antihypertensive medications (Model 3).

In addition, subgroup analyses were performed for age (≥65 years vs. <65 years) and SBP control (≥135 mmHg vs. <135 mmHg). JSH2019 [21] adopted 135/85 mmHg as the diagnostic criterion for hypertension based on HBP. *p*-values < 0.05 were considered indicative of statistically significant findings. Statistical analyses were performed using JMP version 13.2 software (SAS Institute Inc., Cary, NC, USA).

## 3. Results

A total of 1372 consecutive patients with type 2 DM, aged 20–90 years, were recruited for this study. In all, 64 and 422 patients were excluded due to insufficient HBP and UACR data, respectively. In addition, there were 148 patients who were newly prescribed angiotensin II receptor blocker (ARB) or angiotensin-converting-enzyme inhibitor (ACE-I), or who stopped using them during follow-up. Another 263 patients who had moderately or severely increased albuminuria were also excluded. 

**Figure 1:**
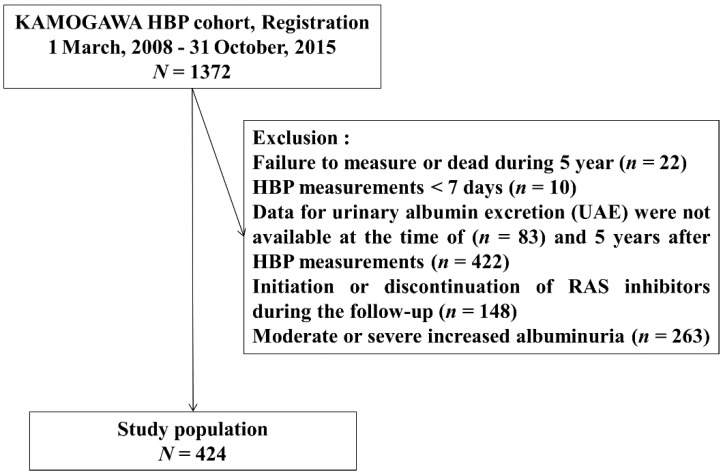
Study flow diagram for the registration of patients.

The final sample included 424 patients with normal or mild albuminuria (Figure 1). Among them, during 5-year follow-up period, 74 patients developed moderately increased albuminuria and 1 patient developed severely increased albuminuria.

Patient baseline demographic and clinical characteristics are presented in Table 1 and Table 2. Median (interquartile range) age, duration of diabetes, BMI, and levels of total cholesterol and those of HbA1C were 64.0 (59.0–70.0) years, 9.0 (4.8–15.0) years, 23.0 (21.4–25.3) kg/m2, 191 (170–212) mg/dL, and 6.6% (6.2%–7.3%), respectively. The patients in the IH-HSBP group were older than those in the high HBP group (69.6 vs. 60.6 years, *p* < 0.001). The unadjusted odds ratio (OR) with 95% confidence interval (CI) of developing diabetic nephropathy, given IH-HSBP, IH-HDBP, and high HBP, was 2.68 (1.36–5.30), 0.78 (0.21–2.81), and 1.63 (0.87–3.04), respectively (Table 3), using normal HBP as a reference. In multivariate analyses, adjusted OR (95% CI) of developing diabetic nephropathy, given IH-HSBP, was 2.36% (1.14%–4.89%, *p* = 0.02) in Model 2 and 2.39% (1.15%–4.96%, *p* = 0.02) in Model 3 (Table 3).

In subgroup analyses, an adjusted OR (95% CI) for developing nephropathy, given IH-HSBP, was 1.68 (0.66–4.27) among age > 65 years (Table 4); meanwhile, in age < 65 years, an adjusted OR (95% CI) was 3.06% (0.63%–15.0%) (Table 4), using normal HBP as a reference.

In subgroup analysis of SBP control, in patients with equal to or more than 135 mmHg, the adjusted odds ratio (95% CI) of IH-HSBP, using normal HBP as a reference group for the development of diabetic nephropathy, was 5.39% (1.92–18.6%) (Table 5). In patients with <135 mmHg, the adjusted odds ratio (95% CI) of IH-HSBP was 0.71% (0.32–1.35%) (Table 5). The odds of each adjusting factor for the development of diabetic nephropathy are presented in Table 6.

## 4. Discussion

In the present study, IH-HSBP was associated with an increased risk of transition to moderate or severe albuminuria in patients with type 2 DM during a 5-year follow-up period. 

The results are in line with the previous 2-year cohort study [8]. The mechanism likely to account for the association between IH-HSBP and diabetic nephropathy risk has been described elsewhere [23,24,25,26,27,28,29]. Increased arterial stiffness has been associated with the development of ISH [30]. Further arterial aging might result in additional increase of IH-HSBP, which is a risk factor for target organ dysfunction [31] and diabetic nephropathy [32]. The association between proteinuria and high BP is strictly related to very high risk of cardiovascular disease in type 2 diabetes [33,34]. In advanced type 2 diabetic nephropathy, appropriate management is of great importance [35]. So, we should adequately man-age home SBP. In HBP management, especially, we should clarify the association be-tween albuminuria and isolated high HSBP.

In the present study, IH-HSBP was associated with an increased risk of diabetic nephropathy; however, high HBP was not. The patients in the IH-HSBP group were older than those in the high HBP group. When arterial stiffness was compared between the IH-HSBP and High-HBP groups using baPWV measurements, there appeared to be higher arterial stiffness among patients in the IH-HSBP group than in those in the High-HBP group (Table S1) [16,36]. Arterial aging in IH-HSBP may be associated with increased odds for the development of diabetic nephropathy. Similarly, the isolated high HDBP group was not associated with an increased risk of diabetic nephropathy development. Those in the isolated HDBP group were younger, had a short duration of diabetes, lower baPWV, and also lower HSBP than the isolated high HSBP group (Table S2). For these reasons, only the isolated HSBP was associated with an increased risk of diabetic nephropathy development in this study.

The effect of IH-HSBP on the development of diabetic nephropathy, defined using estimated glomerular filtration rate (eGFR), is very important. Then, we analyzed the association between IH-HSBP and the development of diabetic nephropathy, defined using eGFR, and found that there was no relationship between them. We examined the association between changes in eGFR and the factors which were associated with IH-HSBP, including duration of diabetes or baPWV, and found no association. We assume that development of diabetic nephropathy, defined using ACR but not eGFR, was associated with pathophysiology of IH-HSBP in this study, although the precise mechanism is not unclear. Moreover, in this study, the mean (standard deviation) change in eGFR over 5 years was −0.37 (7.92) mL/min/1.73^2^, which might be too small to properly analyze the development of diabetic nephropathy.

Initiation or discontinuation of anti-diabetic medications such as sodium glucose co-transporter (SGLT2) inhibitors may affect intra-glomerular pressure and the progression of diabetic nephropathy. However, SGLT2 inhibitors were not used at the start or during the initial 2-year follow-up period of this study. Among 424 patients with type 2 DM in the present cohort, 24 patients were newly prescribed SGLT2 inhibitors during the study period. Nevertheless, use of SGLT2 inhibitors did not affect the risk of diabetic nephropathy associated with IH-HSBP. Most present study patients were prescribed these agents for less than one year, which might have reduced their impact on outcomes of interest. Further studies are needed to examine this effect.

ISH among young-to-middle-aged Japanese people is associated with premature mortality due to cardiovascular disease [37]. In the present study, age-stratified subgroup analysis revealed that the adjusted OR was higher among patients aged <65 years than in those aged ≥65 years. These findings were consistent with those of our previous study [8], in that the association between IH-HSBP and diabetic nephropathy was weakened in patients ≥ 65. Among the patients with IH-HSBP ≥ 65, the progression of diabetic nephropathy was observed in 9.4% (2-year) and 9.5% (5-year). The progression of diabetic nephropathy did not increase over 3 years among the patients with IH-HSBP ≥ 65. Therefore, the association between IH-HSBP and diabetic nephropathy was weakened in patients aged ≥65. Meanwhile, subgroup analyses stratified by SBP status revealed that IH-HSBP increased the risk of diabetic nephropathy only in patients with SBP ≥ 135 mmHg. Patients with IH-HSBP may be at a lower risk if their SBP measurements meet the hypertension diagnostic criteria of less than 135 mmHg [21]. It should be noted that patients in this group were older and more likely to take antihypertensive medications than patients with SBP < 135 mmHg (Table S3). Patients with SBP ≥ 135 mmHg had remarkable ISH, which would be associated with arterial damage and diabetic nephropathy. 

To the best of our knowledge, this is the first study to evaluate the impact of IH-HSBP on the risk of diabetic nephropathy in patients with type 2 DM over the medium to long term. The results support and strengthen previous reports. In addition, the risk of younger patients with ISH was elucidated through the 5-year follow-up period.

Nevertheless, this study has several limitations, which should be considered when interpreting its findings. First, we did not have data on salt intake, protein intake, or levels of exercise, which would be associated with the development of diabetic nephropathy [27,38,39,40,41]. In this regard, we could not clearly identify the prognostic significance of HBP for the development of diabetic nephropathy, even in a longer study. Second, only Japanese men and women were included in the study population. Therefore, these findings might not be generalized to other ethnic groups. Third, only single baseline measurements of BP were performed. This may be potential bias. However, the association of target organ damage was confirmed by BP at baseline or during follow-up [21]. Single BP assessments would be reliable when the addition of subsequent values does not significantly alter the results. Fourth, another important issue is the ultrasound findings on kidneys in the baseline, particularly the size of kidneys, which should be hypertrophic or enlarged before a moderately increased albuminuria development. However, these were not the ultrasound findings on kidneys. Fifth, the risk of ISH, defined by home BP on developing albuminuria in diabetic patients, was similar after the follow-up period and was prolonged for 3 years. The results were essentially similar to previous findings, and thus could not add new information for clinical science. We should at least prolong the follow-up period up to 10 years or more. Finally, a non-albuminuric phenotype has for years been reported in diabetic kidney disease (DKD) of type 2 DM [42]. Therefore, many patients with type 2 DM, despite being normoalbuminuric if they have a GRF of <60 mil/min/1.73m^2^, still have DKD. In the present study, we did not include patients with a GFR of <60 mil/min/1.73m^2^. Thus, we were not able to evaluate the decline in renal function in the definition of DKD in this study. Further studies will be conducted in the future. 

## 5. Conclusions

In conclusion, IH-HSBP in patients with type 2 diabetes mellitus was a prognostic factor for the development of diabetic nephropathy in a prospective 5-year cohort study.

## Figures and Tables

**Table 1:** Characteristics of patients.

Sex	
Male	228 (53.8)
Female	196 (46.2)
Age (y)	64.0 (59.0–70.0)
Duration of diabetes (y)	9.0 (4.8–15.0)
Body mass index (kg/m^2^)	23.0 (21.4–25.3)
Mean morning systolic blood pressure (mmHg)	128.1 (117.4–138.2)
Mean morning diastolic blood pressure (mmHg)	73.2 (66.5–79.9)
Mean evening systolic blood pressure (mmHg)	123.4 (115.0–133.1)
Mean evening diastolic blood pressure (mmHg)	67.7 (61.9–74.2)
Clinic systolic blood pressure (mmHg)	136.0 (123.0–146.0)
Clinic diastolic blood pressure (mmHg)	76.7 (70.0–80.3)
Hemoglobin A1c (mmol/mol)	52.0 (48.6–59.5)
Total cholesterol (mg/dL)	191 (170–212)
Creatinine (mg/dL)	0.70 (0.58–0.83)
eGFR (ml/min/1.73^2^)	75.0 (63.1–89.0)
baPWV	1762 (1501–2002)
Smoking status	
Current smoker	63 (18.1)
Past smoker	109 (31.3)
Alcohol drinking	
everyday	99 (28.6)
social	71 (20.5)
Diabetic complications	
Retinopathy	84 (23.5)
Neuropathy	118 (31.8)
Neuropathy	118 (31.8)
Macrovascular disease	101 (27.0)
Use of antihypertensive medication	192 (45.2)
RAS (−/+)	267/156

For categorical variables, n (%) is presented. For continuous variables, median (interquartile range) is presented. eGFR, estimated glemerular filtration rate; baPWV, brachial–ankle pulse wave velocity; RAS, renin–angiotensin–aldosterone system.

**Table 2:** Characteristics of patients according to the 4 groups based on HBP levels.

Hypertension Status (n)	Normal HBP Group (152)	Isolated High HSBP Group (83)	Isolated High HDBP Group (30)	High HBP Group (159)
Male/female	74/78	40/43	19/11	95/64
Age (y)	64 (58–70)	69 (63–75)	60 (45–65)	63 (58–70)
Body mass index (kg/m^2^)	22.1 (20.9–24.1)	22.5 (21.2–24.7)	23.8 (21.7–26.1)	24.0 (21.8–26.4)
Mean morning systolic blood pressure (mmHg)	115.5 (107.7–119.0	133.3 (128.8–139.6)	120.0 (116.9–122.2)	139.0 (132.2–146.1)
Mean morning diastolic blood pressure (mmHg)	66.3 (62.6–69.0)	69.4 (64.8–72.1)	77.2 (76.4–81.6)	81.6 (77.9–86.7)
Mean evening systolic blood pressure (mmHg)	112.9 (107.6–119.3)	129.6 (123.6–136.3)	118.4 (114.7–123.5)	131.1 (123.7–140.1)
Mean evening diastolic blood pressure (mmHg)	62.9 (58.7–67.2)	63.5 (60.0–67.6)	74.3 (70.9–77.6)	75.1 (70.4–80.6)
Clinic systolic blood pressure (mmHg)	124.1 (114.5–134.8)	141.3 (134.8–151.2)	126.8 (119.0–143.3)	140.7 (130.6–153.0)
Clinic diastolic blood pressure (mmHg)	71.7 (65.8–76.0)	71.7 (65.6–77.7)	82.3 (78.3–92.0)	83.0 (78.7–86.7)
Hemoglobin A1c (%)	6.5 (6.2–7.1)	6.8 (6.2–7.5)	6.4 (6.0–6.8)	6.7 (6.2–7.3)
Total cholesterol (mg/dL)	188.5 (164.8–211.5)	192 (170.5–206)	189 (167–209)	191 (175–216)
Creatinine (mg/dL)	0.67 (0.55–0.80)	0.69 (0.55–0.85)	0.70 (0.62–0.78)	0.70 (0.58–0.83)
eGFR (ml/min/1.73^2^)	77.8 (64.0–95.0)	71.1 (58.0–85.0)	84.5 (76.0–96.8)	75.0 (64.3–86.0)
baPWV (cm/sec)	1584 (1411–1858)	1844 (1645–2059)	1491 (1281–2150)	1726 (1509–1995)
Smoking status (never/past/current)	89/42/20	45/16/21	19/7/4	85/42/27
Alcohol drinking (never/social/everyday)	97/34/20	53/9/20	16/8/6	69/35/49
Retinopathy (NDR/SDR/PDR)	120/13/13	52/18/10	24/4/1	124/19/8
Neuropathy (−/+)	111/41	57/25	28/1	120/37
Macrovascular complication (−/+)	131/21	68/15	29/1	139/20
Antihypertensive medication (−/+)	103/49	39/44	22/8	68/91
RAS (−/+)	116/36	43/40	23/7	85/73

HBP, home blood pressure; HSBP, home systolic blood pressure; HDBP, home diastolic blood pressure; eGFR, estimated glemerular filtration rate; baPWV, brachial–ankle pulse wave velocity; NDR, no diabetic retinopathy; SDR, simple diabetic retinopathy; PDR, proliferative diabetic retinopathy; −, without; +, with. For categorical variables, n is presented. For continuous variables, median (interquartile range) is presented.

**Table 3:** Unadjusted and adjusted odds ratios for the development of diabetic nephropathy.

Hypertension Status (n)	Model 1		* Model 2		* Model 3	
	Unadjusted OR (95%CI)	*p* Value	Adjusted OR (95%CI)	*p* Value	Adjusted OR (95%CI)	*p* Value
Normal HBP group (152)	1		1		1	
Isolated high HSBP group (83)	2.68 (1.36–5.30)	0.004	2.36 (1.14–4.89)	0.020	2.39 (1.15–4.96)	0.019
Isolated high HDBP group (30)	0.78 (0.21–2.81)	0.701	0.54 (0.12–2.53)	0.438	0.54 (0.12–52.5)	0.434
High HBP group (159)	1.63 (0.87–3.04)	0.126	1.57 (0.79–3.12)	0.193	1.60 (0.81–3.17)	0.173

HBP, home blood pressure; HSBP, home systolic blood pressure; normal HBP (morning SBP < 125 mmHg and morning DBP < 75 mmHg); isolated high HSBP (morning SBP > 125 mmHg and morning DBP < 75 mmHg); isolated high HDBP (morning SBP < 125 mmHg and morning DBP > 75 mmHg); and high HBP (morning SBP > 125 mmHg and morning DBP > 75 mmHg). * Model 2: Odds ratios were adjusted for sex, age, duration of diabetes mellitus, body mass index, hemoglobin A1C, total cholesterol, creatinine, and the use of antihypertensive medications. * Model 3: Odds ratios were adjusted for variables in Model 2 and additional adjustment for the use of renin–angiotensin system inhibitors instead of the use of antihypertensive medications.

**Table 4:** Unadjusted and adjusted odds ratios for the development of diabetic nephropathy in patients equal to or more than 65 years old and less than 65 years old.

Hypertension Status	Model 1		* Model 2		* Model 3	
	Unadjusted OR (95%CI)	*p* Value	Adjusted OR (95%CI)	*p* Value	Adjusted OR (95%CI)	*p* Value
≥65 years old						
Normal HBP group	1		1		1	
Isolated high HSBP group	1.90 (0.85–4.23)	0.116	1.70 (0.67–4.33)	0.263	1.68 (0.66–4.27)	0.275
<65 years old						
Normal HBP group	1		1		1	
Isolated high HSBP group	3.08 (0.76–12.5)	0.116	3.07 (0.62–15.1)	0.167	3.06 (0.63–15.0)	0.167

HBP, home blood pressure; HSBP, home systolic blood pressure; * Model 2: Odds ratios were adjusted for sex, age, duration of diabetes mellitus, body mass index, hemoglobin A1C, total cholesterol, creatinine, and the use of antihypertensive medications. * Model 3: Odds ratios were adjusted for variables in Model 2 and additional adjustment for the use of renin–angiotensin system inhibitors instead of the use of antihypertensive medications.

**Table 5:** Unadjusted and adjusted odds ratios for the development of diabetic nephropathy in patients according to systolic blood pressure.

Hypertension Status	Model 1		* Model 2		* Model 3	
	Unadjusted OR (95%CI)	*p* Value	Adjusted OR (95%CI)	*p* Value	Adjusted OR (95%CI)	*p* Value
≥135 mmHg						
Normal HBP group	1		1		1	
Isolated high HSBP group	4.21 (1.73–12.6)	0.0009	5.59 (2.02–19.1)	0.0005	5.39 (1.92–18.6)	0.0008
<135 mmHg						
Normal HBP group	1		1		1	
Isolated high HSBP group	1.31 (0.63–2.52)	0.452	0.75 (0.33–1.57)	0.449	0.71 (0.32–1.35)	0.384

HBP, home blood pressure; HSBP, home systolic blood pressure; * Model 2: Odds ratios were adjusted for sex, age, duration of diabetes mellitus, body mass index, hemoglobin A1C, total cholesterol, creatinine, and the use of antihypertensive medications. * Model 3: Odds ratios were adjusted for variables in Model 2 and additional adjustment for the use of renin–angiotensin system inhibitors instead of use of antihypertensive medications.

**Table 6:** The odds of each adjusting factor for the development of diabetic nephropathy according to systolic blood pressure.

	SBP Control ≥ 135 mmHg	SBP Control < 135 mmHg
Sex	0.86 (0.36–1.98)	0.69 (0.36–1.29)
Duration of diabetes	0.99 (0.95–1.04)	0.98 (0.95–1.01)
Body mass index	1.08 (0.97–1.22)	0.93 (0.86–1.01)
Hemoglobin A1c	1.12 (0.73–1.80)	0.73 (0.52–1.03)
Total cholesterol	1.00 (0.99–1.02)	0.99 (0.98–1.003)
Creatinine	1.13 (0.51–6.26)	0.74 (0.13–4.56)
Use of antihypertensive medication	0.90 (0.38–2.06)	0.69 (0.37–1.32)
Use of RAS	1.00 (0.43–2.29)	0.78 (0.40–1.56)

SBP, systolic blood pressure; RAS, renin–angiotensin–aldosterone system.

## Data Availability

Data are available upon reasonable request to the corresponding author.

## Supplementary Materials

The following are available online at https://www.mdpi.com/article/10.3390/jcm10091929/s1, Table S1: The comparison of arterial stiffness among patients with isolated high HSBP or High-HBP, Table S2: The comparison of the patients with isolated high HSBP or isolated high HDBP, Table S3: The comparison of patients according to systolic blood pressure.
