# Supplementary material for: Impact of Isolated High Home Systolic Blood Pressure and Diabetic Nephropathy in Patients with Type 2 Diabetes Mellitus: A 5-Year Prospective Cohort Study
Source: J Clin Med. 2021 Apr 29;10(9):1929. doi: 10.3390/jcm10091929 (PMC8124698; doi:10.3390/jcm10091929)
Supplement: Supplementary file 1 [file jcm-10-01929-s001.zip › jcm-1165655-supplementary.pdf]

Supplementary files

Table S1. The comparison of arterial stiffness among patients with isolated high HSBP or High-HBP.

	isolated high HSBP	High-HBP	*P* Value
baPWV (cm/sec)	1844 (1645-2059)	1726 (1509-1995)	0.053

Data was expressed as median (interquartile range). The difference between two group was evaluated by Manne-Whitney U test. HBP, home blood pressure; HSBP, home systolic blood pressure; baPWV, brachial ankle pulse wave velocity.

Table S2. The comparison of the patients with isolated high HSBP or isolated high HDBP.

	Isolated high HSBP	Isolated high HDBP	*P* Value
Age (year)	69 (63-75)	60 (45-65)	<0.01
Duration of diabetes (year)	11 (7-20)	8 (4-11.3)	<0.01
HSBP (mmHg)	133.3 (128.8-139.6)	120.0 (116.9-122.2)	<0.01
baPWV (cm/sec)	1844 (1645-2059)	1491 (1281-2150)	<0.01

Data was expressed as median (interquartile range). The difference between two group was evaluated by Manne-Whitney U test. HBP, home blood pressure; HSBP, home systolic blood pressure; baPWV, brachial ankle pulse wave velocity.

Table S3. The comparison of patients according to systolic blood pressure.

	SBP ≥135 mmHg	SBP <135 mmHg	*P* Value
age	66.2	61.6	P< 0.01
Use of antihypertensive medications (%)	69.1	45.5	P< 0.01

Data was expressed as mean or %. The difference between group was evaluated by ManneWhitney U test. SBP, systolic blood pressure.
